# Gaseous Enemata

**Published:** 1887-08

**Authors:** 


### *GASEOUS ENEMATA.*

Dr. E. T. Bruen, of Philadelphia, read a paper before the Climatological Association, on the above subject, on the treatment of consumption:

Of the therapeutic measures under the control of the profession, diet, climate and suitable hygiene are of principal importance. It is possible by clymatic and dietetic treatment to so change the nature of the tissues that they shall not be suitable culture media for the growth of the bacillus of tuberculosis. I wish, however, to refer more particularly to the use of the Bergeon method by the injection of sulphuretted hydrogen. To antagonize the specific cause of the disease, this method has been a failure so far as my experience goes. Since February last I have had under treatment sixty-one cases by this method. Systematic examinations of the sputa have been made by Dr. E. O. Shakespeare. There has been no apparent reduction in the number or change in the character of the bacillus. This method should be classed among the methods at our disposal for the treatment of this disease. The good effects in my hands have been reduction of temperature, reduction of expectoration, very often a complete suppression of bronchial catarrh, and relief of cough. This leads to improved digestion and enables the dietetic treatment to be carried out with great thoroughness. Forty-four of the cases showed improvement to a certain extent, the average gain in flesh being about five pounds. In one-half the cases the temperature has been brought to the normal, while the remainder, although the temperature has not been brought to the normal, it has been reduced two or three degrees. In fifteen cases the results have been negative, but in no case did any harm follow the use of this plan of treatment.

The improvement has been most marked where there is a considerable catarrhal element. Those cases in which there has been more or less thickening of the lung, with the general symptoms well marked, wasting, loss of flesh and weight without much rise of temperature, I have found not specially benefitted by the injection of gas. I have had the opportunity of making a *post mortem* in one case which had been subjected to this treatment. Although the cavities in the lungs were unusually clean, I did not observe any evidences of cicatrization. With reference to the strength of the solution, I have not found strong solutions at all satisfactory. The best results have been obtained from a solution of 5 grains of sulphide of sodium with 5 grains of chloride of sodium in 1½ pints of water. I have never found it desirable to administer more than 1½ gallons of gas at one time. I insisted that the injection be made slowly and that one-half to three-quarters of an hour should be occupied. I have not derived as much satisfaction, in the treatment of the various forms of phthisis to which I have referred, from any method as I have from the injection of sulphuretted hydrogen. I have always tested the breath for the presence of sulphuretted hydrogen. I have had negative results in at least eight out of every ten cases.—*American Medical Journal.*

